# Influences of pH on transport of arsenate (As^5+^) through different reactive media using column experiments and transport modeling

**DOI:** 10.1038/s41598-020-59770-1

**Published:** 2020-02-26

**Authors:** Srilert Chotpantarat, Chonnikarn Amasvata

**Affiliations:** 1 Department of Geology, Faculty of Science, Chulalongkorn University, Bangkok, 10330 Thailand; 2 International Postgraduate Programs in Environmental Management, Graduate School, Chulalongkorn University, Bangkok, Thailand; 3 Research Program of Toxic Substance Management in the Mining Industry, Center of Excellence on Hazardous Substance Management (HSM), Chulalongkorn University, Bangkok, Thailand; 4 Research Unit of Green Mining (GMM), Chulalongkorn University, Bangkok, Thailand

**Keywords:** Environmental chemistry, Hydrology

## Abstract

This research aims to evaluate the effects of pH, including both acidic and neutral conditions to simulate an acid mine environment, on the sorption and transport of As(V) in contaminated groundwater through different reactive materials by using column experiments and mathematical modeling. Six saturated columns were set up to evaluate the migration and removal efficiency of As(V) with three different materials acting as permeable reactive barrier (PRB) media under different pH conditions (pH 4 and pH 7). The reactive materials consisted of pure sand (control column), iron oxide-coated sand (IOCS) and a combination of IOCS and zero-valent iron-coated sand (ZVICS) (ZVICS + IOCS). According to the column experiments, the descending order of removal capacity (mg As/g) for ZVICS + IOCS, IOCS and sand was 0.452 > 0.062 > 0.0027 mg As/g at pH 4 and 0.117 > 0.0077 > 0.0022 mg As/g, respectively, at pH 7. The column experiments showed that the removal and retardation factor (*RF*) of As(V) generally increased with decreasing pH. The SEM images and the corresponding EDX spectra of acid-washed natural sand, IOCS and ZVICS + IOCS from the columns showed that the peak of As was detectable on the reactive materials. The mechanism of As(V) sorption onto sand at pH 4 and pH 7 corresponded to the uniform (equilibrium) solute transport model, whereas the IOCS and ZVICS + IOCS columns corresponded to the two-site model (TSM) with the Freundlich isotherm. The fraction of instantaneous sites (*f*) for As(V) sorption onto IOCS and ZVICS + IOCS appeared to decrease with increasing pH, especially for ZVICS + IOCS, which indicates that nonequilibrium sorption/desorption mainly dominated during As(V) migration.

## Introduction

Arsenic (As) can contaminate the environment via natural processes (e.g., erosion and weathering processes of soils, minerals, and ores) and human activities (e.g., mining activities, combustion of fossil fuels, and use of pesticides and herbicides)^1^. Arsenic is often found at elevated concentrations in groundwater. Its occurrence in groundwater depends on the types of rock and soil in the area. Regarding the increased concentrations of As in natural groundwater, there are many areas in the world, including some areas in Thailand, where As is found in higher concentrations than the World Health Organization (WHO) guideline of 10 μg/L^2^. For example, Choprapawon *et al*.^3^ has studied the impact of As for decades in the Ronpibool District, Nakorn Sri Thammarat, the Southern province of Thailand. The provincial administrative organization and the inhabitants of this district have recognized the harmful effects of As contamination in natural water sources in Ronpibool District for more than 10 years. Moreover, villagers who live near the area surrounding gold mines in the Wangsaphung District, Loei province, Thailand, complained in 2006 about contamination of As and found significant high levels of As in soils, water and plants near the gold mine area^4^. This has become a serious issue because groundwater has been increasingly used for drinking water production in many countries.

Arsenic contamination in drinking water causes serious short- and long-term adverse effects on the health of humans and other living organisms. Short-term exposure (over days or weeks) to high levels of As in drinking water can result in nausea, diarrhea, and muscle pain. Moreover, long-term exposure (over years or decades) to high levels of As in drinking water can result in skin changes, damage to major body organs and some types of cancer^1^. High concentrations of As are found in groundwater in both oxidizing and reducing conditions. The presence of As in oxidizing conditions generally occurs in the form of As(V), whereas As in reducing conditions mainly occurs in the form of As(III). In the last decade, the problem of elevated As concentrations in Thailand has usually occurred near mining areas^5–8^. Under the generally acidic conditions near a mining area, a high concentration of As combined with other elements is commonly found in lethal amounts. The oxidation of arsenopyrite (FeAsS) leads to sulfuric acid and the release of As in the unsaturated zone, which is the source of acid mine drainage (AMD). In general, As(III) can be released by mining activities and then oxidized to As(V) by exposure to O_2_ in unconfined shallow aquifers. Therefore, in this study, we focused on As(V) removal since As(III) can always be oxidized to As(V) by a variety of treatment processes.

This study focused on treating As(V) that occurs in unconfined shallow aquifers. Zero-valent iron (ZVI) is a good quality reactive material that is mostly used as a permeable reactive barrier (PRB) for the remediation of contaminants in groundwater^9^. Mak *et al*.^10^ recently found that ZVI has a high removal capacity for As contamination in water. However, ZVI can be oxidized by water itself. Reactions may occur associated with the reduction of water or nitrates to produce gases, such as hydrogen or nitrogen^11–13^. As a result, the precipitation of solids and the formation of gases from the oxidation and reduction of ZVI can drop the permeability of a PRB and cause hydraulic failure. Although ZVI has disadvantages, it has an advantage when mixed with iron oxide-coated sand (IOCS)^14^. Mak *et al*.^10^ studied the removal of coexisting Cr(VI) and As(V) by using a combination of zero-valent iron (Fe°) and IOCS in batch testing. They found that using a mix of Fe° and IOCS as a reactive material has a high removal efficiency and could be better used in PRB for Cr(VI) and As(V) removal from contaminated groundwater.

Recently, innovative technologies, such as coating iron-oxide onto sand surfaces, have become of interest because they are commonly inexpensive and have high efficiencies for the removal of heavy metals from contaminated water (i.e., Cr(VI), Pb(II), Cu(II), As(III))^15–18^. Past research on the use of individual reactive materials such as granulated iron hydroxide (GIH), ferrihydrite-coated sand, IOCS, sulfate modified iron oxide-coated sand (SMIOCS) and ZVI^16–24^, for the removal of heavy metals has mainly focused on batch experiments^25^ rather than flow-through systems or column experiments that can be applied for *in situ* remediation, such as the permeable reactive barrier (PRB) technique. Furthermore, Das *et al*.^26^ found that three ZVI materials can be used to effectively remove Se(VI) in batch testing and the process is influenced by other anions, SO_4_^2−^ and NO_3_^−^. The removal rate can be explained by first-order reaction kinetics. Some studies have used ZVI (e.g., granular ZVI, cast iron) as reactive media in column studies, but they observed precipitation of solids and gas generation, resulting in gas clogging and decrease of the hydraulic conductivity and residence time in the column experiments^13,27,28^. To prevent hydraulic failure in the PRB system, in this study, ZVI-coated sand (ZVICS) was synthesized and investigated instead of ZVI. Such batch experiments cannot thoroughly explain the effects of water flow through reactive media on As(V) removal and migration (e.g., IOCS and/or zero-valent iron-coated sand (ZVICS)), as normally occurs in column experiments with simulated PRBs. As mentioned, although there are already some works about permeable reactive barriers and As removal in the literature, they are still limited; furthermore, most As adsorption studies have focused on batch mode, and most previous studies did not apply mathematical modelling to explain sorption and migration behavior through the columns^19,29–32^, so the scale up and modeling of adsorption columns is of interest. Thus, to obtain a better understanding of the removal and transport of As(V) by different reactive media in acidic mine drainage, in the current study, HYDRUS-1D, including an equilibrium model and a chemical nonequilibrium model, was used to explain the sorption and transport mechanisms of As(V) in the solution by reaction with various reactive media. As mentioned above, there are a few studies on the removal and transport of As(V) by IOCS in combination with ZVICS using column experiments and transport modeling. Therefore, an investigation into the reactions among As(V), IOCS, and ZVI using column experiments and modeling is crucial and can be further applied in real site remediation. Overall, this study aimed to (a) evaluate As(V) sorption, migration, and removal efficiency in contaminated water with combinations of IOCS and ZVICS, (b) describe the effects of pH conditions on the mechanisms of As(V) migration through columns with different reactive materials, and (c) ascertain the appropriate sorption and transport parameters of equilibrium and/or chemical nonequilibrium models to further explain As(V) transport in different reactive media under acidic mine drainage. The research was conducted with three different reactive materials under two different pH values (pH 4 and pH 7), comprising six columns. The reactive materials used to compare As(V) removal efficiency in column experiments consisted of pure sand (control column), IOCS, and a combination of ZVICS and IOCS (ZVICS + IOCS). Our final findings could be further applied as predictive framework for the *in situ* site remediation of groundwater contaminated with As(V) in unconfined aquifers in acidic mine environments.

## Materials and Methods

### Reagents

The background stock solution was prepared by dissolving NaNO_3_ mixed with NaOAc in deionized (DI) water and controlling the ionic strength at approximately 0.02 mM. The solution was then adjusted with 0.01 HNO_3_ until the pH reached 4 or 7 (±0.2). Individual As(V) stock solutions (10 mg/L) were prepared by dissolving appropriate amounts of sodium hydrogen arsenate (HAsNa_2_O_4_·7H_2_O) in the background solution at pH 4 and 7.

### Sorbent preparation: sand, IOCS, and ZVICS

#### Sand

The grain diameter of the quartz sand ranged from 0.6–0.8 mm. The sand was cleaned by ultrasonication in 0.01 M NaOH for 30 min and rinsed with DI water. Then, it was dried in a hot-air oven at 105 °C for 24 hrs and stored in a plastic container with silica gel.

#### Iron oxide-coated sand (IOCS)

The IOCS preparation method followed the study of Dhagat *et al*.^15^. After acid washing, the oven-dried sand was added to 1 M FeCl_3_ solution. The pH was adjusted by adding 6 M NaOH until reaching a pH of approximately 8–9, and then the solution was kept for 24 hrs. The coated sand was rinsed with DI water and oven dried at 105 °C for 5–6 hrs. The coated sand was stored in a plastic container with silica gel^15^.

#### Zero-valent iron-coated sand (ZVICS)

The zero-valent iron was synthesized by adding 0.75 M NaBH_4_ dropwise (approximately 40–50 drops/min) into 0.135 M FeCl_2_·6H_2_O in a water bath until the pH was 6. The washed sand was submerged in the mixed solution. The mixture was shaken in a water bath at 200 rpm for 4 hrs while the temperature was maintained at 60 °C. The sand coated with ZVI was washed at least 3 times with 99% ethanol and purged with N_2_ to prevent oxidation. The ZVI-coated sand was stored in a dark plastic container and kept in a desiccator.

The material structures of the reactive materials (sand, IOCs and ZVICS) were analyzed by X-ray diffraction analysis (XRD) using a Bruker AXZ, Germany, model D8 Advance-X-ray diffractometer at the Department of Geology, Faculty of Science, Chulalongkorn University. The X-ray diffractometer used copper Kα radiation (λ = 1.5404 Å) with a scan rate of 2°/min and a step of 0.02° over Bragg angle ranging between 10–60°. The chemical properties of the sand, IOCS and ZVICS were analyzed by X-ray fluorescence spectrometry (XRF) using Bruker model S8 Tiger spectrometer. The point of zero charge (pHpzc) was investigated by two titration methods^33^. The specific surface area of the reactive materials was determined by the Brunauer, Emmett and Teller (BET) method^34^ via nitrogen adsorption-desorption isotherms using a surface area analyzer (BELSORP-mini, BEL Japan, Inc.).

### Column experiments

Six columns were packed with the different reactive materials (sand, IOCS, and ZVICS mixed with IOCS) under acidic (pH 4) and neutral (pH 7) conditions. Acrylic columns with a 2.5 cm inner diameter and 10 cm length were used in the column experiments. The columns had an effective porosity varying from 0.37–0.38 and a bulk density varying from 1.51–1.53 g/cm^3^ (Table 1). The influent was injected at the bottom of the column with an average linear velocity of approximately 2.30 m/day (or 0.267 cm^3^/min). The effect of pH on the removal of As(V) in each column was investigated as shown in Table 1.

**Table 1:** As sorption and transport with different reactive materials (sand, IOCS, ZVICS-IOCS) through saturated columns for solution pH values of 4 and 7.

Column Nos.	Reactive materials	pH	Bulk density (g/cm^3^)	Porosity(−)	Pore volume (cm^3^)	Seepage velocity (m/day)
1	Sand	4.0 ± 0.1	1.510	0.3772	19.10	2.07
2	IOCS	4.0 ± 0.1	1.518	0.3708	18.78	2.07
3	ZVICS-IOCS	4.0 ± 0.1	1.524	0.3658	18.53	2.07
4	Sand	7.0 ± 0.2	1.509	0.3768	19.08	2.07
5	IOCS	7.0 ± 0.2	1.529	0.3705	18.76	2.07
6	ZVICS-IOCS	7.0 ± 0.3	1.523	0.3659	18.53	2.07

Each column test was conducted separately, but each column experiment was reproduced column-to-column in a similar manner, and a similar bulk density was maintained. Moreover, each column was treated with a similar average linear velocity of 2.30 m/day. Our previous study^35^ used columns prepared with the same technique, the so-called wet packing method^36–38^, and the dispersivity of each column (~1.07 cm) was quite similar. Furthermore, to assure the reproducibility of the column experiment, another previous study^39^ duplicated the column test, performed with the wet packing technique, to investigate Ni and Pb transport; the retardation factor (RF) and sorbed metal values in the duplicate columns provided similar results. As mentioned, it would be possible to apply this technique to reproduce the column study.

The columns were equilibrated by flushing several pore volumes (PVs) of DI water. As(V)-free background solutions with constant pH values of ~4.0 and ~7.0 were used to flush the column for at least 3–4 PVs to establish steady state flow and standardize the chemical conditions. As(V) solutions with concentration of 10 mg/l and different pH conditions (4 and 7) were then pumped into the bottom of the column via a piston pump at a constant velocity of approximately 2.30 m/day (see Table 1). When the maximum relative concentration of As(V) (Ci/C0) was approximately 1.0, several PVs of background solution with the same pH conditions were applied to the column to ensure that there was no As(V) in the effluent. The effluent was collected in tubes by using a fraction collector at regular time intervals and measured by inductively-coupled plasma mass spectrometry (ICP-MS). The breakthrough curves (BTCs) of As(V) are represented with respect to the relative pore volume (V_i_/V_0_) and concentration of As(V) (C_i_/C_0_). Scanning electron microscope and energy dispersive X-ray spectrometer (SEM-EDS) of the coated sand were performed using a JEOL scanning microscope (JEOL, JSM-6610LV) to analyze the surface features and elemental compositions of the surface mineral deposits on the media coatings. The coated sand samples were air dried, glue mounted and covered with gold film by vacuum electric arc prior to instrumental analysis.

### Retardation factor (*RF*)

For comparison, the adsorption efficiencies were determined from the retardation factor (*RF*) calculated by the area method^40^. The equation for calculating the retardation factor (*RF*) is shown in Eq. 1:

1 $$RF=P{V}_{1}-\mathop{\sum }\limits_{i=0}^{P{V}_{1}}(\frac{{C}_{i}}{{C}_{0}})\Delta PV$$

where *C*_*i*_ is the concentration of As(V) in the effluent at time i

*C*_*0*_ is the initial concentration of As(V) in the influent

*PV*_*1*_ is the number of pore volumes at a relative concentration (*C*_*i*_*/C*_*0*_) of As(V) = 1.0.

*ΔPV* is the difference in volume of successive values of *PV* (*PV*_*i+1*_ − *PV*_*i*_)

### Hydrus-1D model

The Hydrus-1D model can be used to simulate water flow and the migration of heavy metals in variably saturated porous media^41^. The model can be applied for different physical equilibrium and chemical nonequilibrium flows and migration in both direct and inverse models^39^.

The two-site model is a type of chemical nonequilibrium model. This model can be expanded by assuming that the sorption sites can be divided into two fractions^42,43^. One fraction of the sorption sites is assumed to be instantaneous, whereas the second section is assumed to undergo kinetic sorption. The two-site model (TSM) considers nonequilibrium sorption-desorption reactions. This model can be expanded in terms of Eq. 2–5 as follows:

2a $$\frac{{\rm{\partial }}\theta c}{{\rm{\partial }}t}+\rho \frac{{\rm{\partial }}{S}^{e}}{{\rm{\partial }}t}+\rho \frac{{\rm{\partial }}{S}^{k}}{{\rm{\partial }}t}=\frac{{\rm{\partial }}}{{\rm{\partial }}z}(\theta D\frac{{\rm{\partial }}c}{{\rm{\partial }}z})-\frac{{\rm{\partial }}qc}{{\rm{\partial }}z}\pm {[\frac{{\rm{\partial }}c}{{\rm{\partial }}t}]}_{rxn}$$

2b $${s}^{e}=f\,{K}_{d}c$$

2c $$\rho \frac{\partial {s}^{k}}{\partial t}=\alpha \,\rho ({s}^{e}-{s}^{k})-{\varnothing }_{k}$$

2d $${s}^{k}=(1-f){K}_{d}c$$

where

*f* is the fraction of exchange sites assumed to be in equilibrium with the liquid phase, -

*α* is a first-order kinetic rate constant, T^−1^

*c* is the As(V) concentration, M L^−3^

*s*^*e*^ is the As(V) concentration sorbed on instantaneous sites, M M^−1^

*s*^*k*^ is the As(V) concentration sorbed on time-dependent or kinetic sites, M M^−1^

*t* is the time, T

${\varnothing }_{k}$ is a sink-source term at the kinetic sorption sites, M L^−1^T^−1^

${K}_{d}$ is the distribution coefficient and can be replaced by Freundlich and Langmuir parameters, LM^−1^

The mechanism of As(V) sorption onto a reactive material is presumably equilibrium sorption when the fraction sites have a value of ~1.00. However, if the fraction sites have a value much less than 1.00, it can be assumed that the mechanism of As(V) sorption onto the reactive material is nonequilibrium two-site sorption. The results from the column experiment were analyzed by using Hydrus-1D model (equilibrium model, chemical nonequilibrium model, or two-site model, TSM) to describe the main mechanisms controlling As(V) transport in the various reactive materials in the saturated columns (sand, IOCS, and ZVICS-IOCS). The experimental data were fitted with equilibrium and chemical nonequilibrium models. For the equilibrium model, the fitted sorption parameters were the Freundlich constants (*Kf* and *1/n*). Additionally, the chemical nonequilibrium TSM model was applied to estimate the nonequilibrium parameters, including *Kf*, *1/n*, *f* and *α* for As(V) transport in the column experiments. The sum of square errors (SSE) and coefficient of determination (R^2^) were used to assess the goodness of fit the curves^44^.

## Results and Discussion

### Chemical properties of sand, IOCS, and ZVICS

The XRF results show that the main chemical component of the sand was SiO_2_. In addition to SiO_2_ (99.50%), the sand primarily consisted of Fe_2_O_3_ (0.24%) and Al_2_O_3_ (0.10%); IOCS consisted of SiO_2_ (99.30%), Fe_2_O_3_ (0.38%) and Al_2_O_3_ (0.11%); and ZVICS mostly consisted of SiO_2_ (99.40%) and Fe_2_O_3_ (0.43%). The BET areas of the sand, IOCS and ZVICS were 0.049, 0.78 and 0.81 m^2^/g, respectively. The BET results for IOCS and ZVICS show increases since the irregular structure of the coated particles on the sand grains caused an increased surface area. Bakather *et al*.^44^ found that the removal of selenium (Se) by CNTs modified with 5%, 10% and 20% iron oxide nanoparticles increased due to increasing available sorption area, with apparent values of 226.6, 295.4, and 360 m^2^/g, respectively. Furthermore, Sharma *et al*.^45^ found that the iron sorption capacity onto coated sand appeared to be 10 to 55 times higher than that of the new sand due to the large specific surface area. However, the chemical composition, crystal structure and surface properties of the surface coating were also influenced by the sorption of iron as well. The PZCs of quartz sand, IOCS and ZVICS were 5.0, 7.0 and 7.5, respectively. The PZC values of the reactive media influence the surface charge behavior and consequently electrostatic attraction of adsorbates, such as As(V). Moreover, the XRD data indicated that both reactive materials (IOCS and ZVICS) were different (see Supplementary Information Figs. SI.1 and SI.2). The results of the XRD analysis supported that the sand was a pure quartz sand, showing prominent peaks at 26.43, 49.75, 59.13 and 67.65° (2$\theta$). The peak at 26.43° was dominant, with the highest intensity. The mineral type of IOCS was identified as goethite (Fe_2_O_3_ H_2_O), showing prominent peaks at 21.09, 33.15, 36.65 and 53.21° (2$\theta$). Moreover, the mineral type of ZVICS was identified as a mixture of iron silicide (Fe_2_Si) and synthetic irons (Fe), showing peaks at 45.60, 66.44, 84.20 and 101.67° (2$\theta$) for Fe_2_Si and at 44.67, 65.02 and 82.33 for Fe. The results of the XRD patterns indicated that the reactive materials were IOCS and ZVICS.

### Effect of pH on As(V) in the water-saturated sand columns

**Figure 1:**
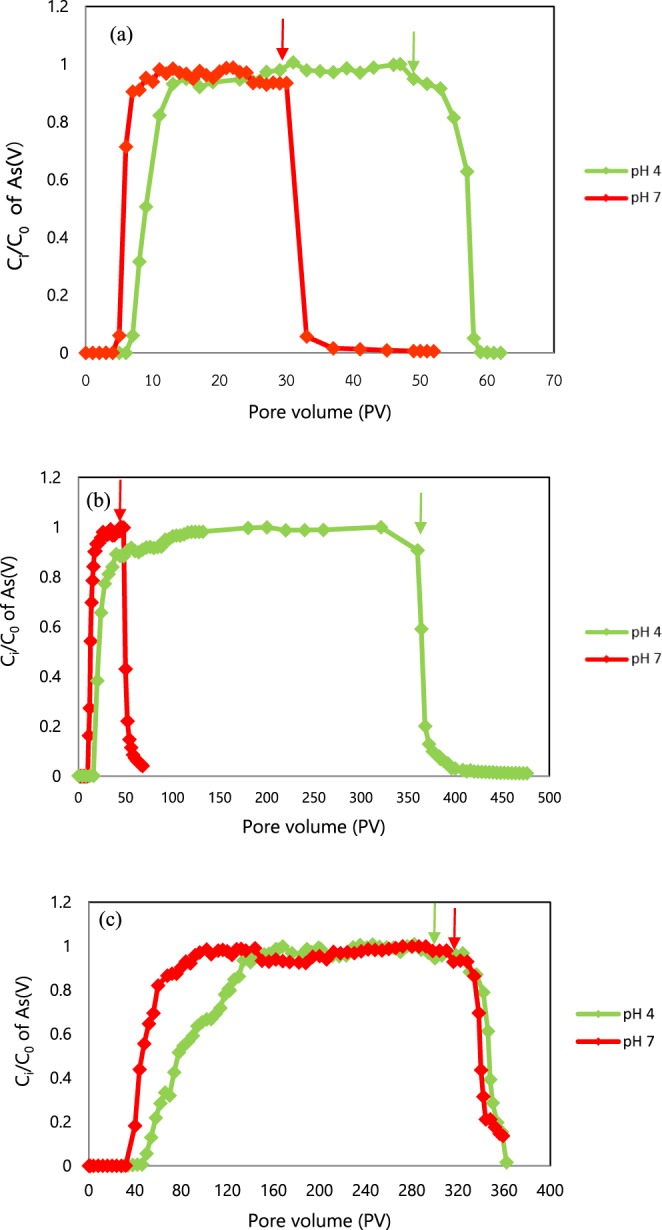
Breakthrough curves of As(V) at pH 4 and pH 7 in (**a**) the water-saturated sand column, (**b**) the IOCS columns, and (**c**) the IOCS mixed with ZVICS columns. Injection of background solution indicated by arrows.

As(V) solutions were injected into the water-saturated columns under different pH values of 4 and 7 to evaluate the migration of As(V) as illustrated in Fig. 1. In the sand column at pH 4, the maximum relative concentration (C_i_/C_0_) of As(V) was 1 at approximately 31 PVs, whereas for the sand column at pH 7, it was 1 at approximately 11 PVs. After surpassing breakthrough at Ci/C0=1, several PVs of background solution were flushed at approximately 30 PVs and 49 PVs for the sand columns for pH 4 and pH 7, respectively (Fig. 1(a)). Then, the breakthrough curve (Fig. 1(a)) showed a sudden decrease in As(V) concentration. Furthermore, the complete breakthrough curves for sand, IOCS, and IOCS-ZVICS columns (Fig. 1) consisting of both rising and declining limbs is generally used to explain whether the sorption and desorption processes occur via equilibrium or chemical nonequilibrium, as presented in section 3.6.

The retardation factor (*RF*) and removal capacity of As(V) during transport through the water-saturated sand columns at pH 4 and 7 decreased from 10.17 to 6.03, and the removal capacity decreased from 0.0027 to 0.0022 mg As(V)/g sand, respectively (see Table 2). Therefore, the removal capacity of As(V) onto sand at low pH was slightly higher than that at high pH^46^. Moreover, according to the mass titration curve of quartz sand, the pH_pzc_ of quartz sand was approximately 5.0, which caused the column at pH 4 to have a more positive charge on the sand surface, whereas the sand surface column at pH 7 appeared to have a higher negative charge. In other words, this result showed that As(V) sorption onto the water-saturated sand column increased with decreasing solution pH because decreasing the pH of the solution below the PZC of the quartz sand can increase the number of available sorption sites to sorb As(V) in the sand columns. As a result, the sorption of As(V) onto the sand column at pH 4 was better than that at pH 7, which agreed with the retardation factor (*RF*) data.

**Table 2:** Retardation factor (*RF*) and As(V) removal of columns containing different reactive materials at different solution pH values.

Column experiment (No.)	Reactive materials	pH	Initial As(V) conc. (ppm)	Mass of reactive materials	*RF*	*As(V) Influent (mg)	*As(V) Effluent (mg)	As(V) Removal (mg/g)
1	Sand	4.0 ± 0.1	10.71	74.25	10.17	5.31	3.47	0.0248
4	Sand	7.0 ± 0.2	9.58	74.13	6.03	1.99	1.06	0.0125
2	IOCS	4.0 ± 0.1	9.34	74.57	37.13	17.94	11.79	0.0825
5	IOCS	7.0 ± 0.2	8.26	74.80	13.78	3.41	1.75	0.0222
3	ZVICS-IOCS	4.0 ± 0.1	9.73	74.80	87.24	21.64	11.83	0.1311
6	ZVICS-IOCS	7.0 ± 0.3	8.77	74.79	67.33	23.04	14.27	0.1173

*Calculated from the complete breakthrough curves.

### Effect of pH on As(V) sorption in the IOCS columns

As(V) solutions were injected into the IOCS columns under different pH values of 4 and 7 to elucidate migration of As(V) as illustrated in Fig. 1(b). The breakthrough curves of As(V) transported through the IOCS column at each pH showed the relationship between the relative concentration (C_i_/C_0_) of As(V) and pore volume (PV). The time intervals needed to reach the maximum relative concentration (C_i_/C_0_ ~1.0) of As(V) in each column were different. For the IOCS column at pH 4, the maximum relative concentration of (C_i_/C_0_) As(V) was 1 at approximately 116 PVs, whereas at pH 7 the maximum relative concentration of (C_i_/C_0_) As(V) was 1 at approximately 26 PVs.

The retardation factor (RF) and removal capacity of As(V) solutions with pH 4 and pH 7 that migrated through the IOCS column decreased from 37.13 to 13.78, respectively, and the removal capacity decreased from 0.0621 to 0.0077 mg As(V)/g, respectively. Similarly, Gupta *et al*.^47^ studied sorption of As(III) on IOCS in batch studies and found that the sorption capacity of the coated sand was higher than the uncoated sand, from 0.0056 mg/g to 0.0286 mg/g. According to the study of Garrido-Hoyos and Romero-Velazquea^24^, silica sand coated with Fe(III) had increased surface sites and sorption capacity for metalloid (As).

The results illustrated that As(V) sorption decreased with increasing pH value, which agrees with the study of Hsu *et al*.^23^, who conducted batch sorption experiments of As(V) onto IOCS with a particle size ranging from 0.7–0.75 mm. The PZC of the IOCS was approximately 7 ± 0.4. They found that the Langmuir model could well explain the sorption capacity of As(V) at pH 5, pH 6, pH 7 and pH 8, with values in the descending order of 0.022 mg/g, 0.0215 mg/g, 0.021 mg/g and 0.0175 mg/g, respectively. For IOCS at pH 7, the removal capacity results from the column experiments in this current study (0.008 mg/g) were 2–3 times less than the sorption capacity (0.021 mg/g) from the study of Hsu *et al*.^23^, perhaps because of the different techniques (batch experiments v.s. column experiments) used to estimate the sorption capacity as well as the properties of the IOCS (specific surface area and As/Fe ratio) used in their study. The removal of As(V) in the column experiment was performed in hydrodynamic conditions including a water velocity of 2.3 m/day, causing the sorption to not reach equilibrium conditions. This caused the sorption efficiency to be lower than that obtained under batch conditions. Ko *et al*.^48^ found that the sorption coefficient of As(V) onto colloidal iron oxide-coated sand (CIOCS) in a batch study (1.30 L/kg) was lower than that obtained in a column study (0.023–0.85 L/kg) due to the incomplete sorption mechanism. Moreover, Daus *et al*.^19^ found that the sorption capacity of As(V) when using GIH in column experiments (2.3 mg/g) was 2.3 times lower than that in batch experiment (5.2 mg/g) due to preferential flow in the small-scale column experiment. The ratio of sorbed As and Fe can be used to assess the available sorption sites for As and to explain the removal efficiency of As(V)^48^. In the current study, the ratio of As to Fe appeared to be constant.

Moreover, according to the results of the mass titration curve, the pH_pzc_ of the IOCS was approximately 7.0, which caused the column of pH 4 to be more positively charged on the surface, whereas at pH 7 the surface appeared to be neutrally charged. As a result, the positive charge of IOCS may increase the removal of H_2_AsO_4_^−^ by sorption^48^. Moreover, the *RF* of the IOCS column at pH 4 was 37.13, which was higher than that of the IOCS column at pH 7 (13.78).

### Effect of pH on As(V) sorption in the combined ZVICS-IOCS column

As(V) solutions were injected into the ZVICS-IOCS columns under different pH values of 4 and 7 to elucidate the migration of As(V) illustrated in Fig. 1(c). The breakthrough curves of As(V) transported through the ZVICS-IOCS columns in each pH showed the relationship between the relative concentration (C_i_/C_0_) of As(V) and pore volume (PV). The time intervals needed to reach the maximum relative concentration (C_i_/C_0_ ~1.0) of As(V) in each ZVICS-IOCS column were different. For the combined ZVICS-IOCS column at pH 4, the maximum relative concentration (C_i_/C_0_) of As(V) was 1 at approximately 142 PVs, whereas the maximum relative concentration (C_i_/C_0_) of As(V) was 1 at approximately 96 PVs for the combined ZVICS-IOCS column at pH 7. The retardation factor (*RF*) and sorption capacity of As(V) at pH 4 and pH 7 during transport through the ZVICS-IOCS combination column decreased from 87.24 to 67.33, and the removal capacity decreased from 0.131 to 0.117 mg As(V)/g, respectively.

The results showed that the As(V) removal decreased with increasing pH, which is similar to the results for the sand and IOCS columns. Additionally, the results for the retardation factor (*RF*) and removal capacity (mg/g) of ZVICS combined with IOCS were higher than those of the IOCS and sand columns (*RF*_ZVICS-IOCS_ > *RF*_IOCS_ > *RF*_sand_ and Q_ZVICS-IOCS_ > Q_IOCS_ > Q_sand_). It can be concluded that the mixing of ZVICS and IOCS can increase the removal capacity of As(V) compared with using IOCS and sand packed in columns only. The retardation factors (*RF*) and removal capacities can be summarized as:

**At pH 4**

$${{\rm{RF}}}_{{\rm{sand}}}(10.17) < {{\rm{RF}}}_{{\rm{IOCS}}}(37.13) < {{\rm{RF}}}_{{\rm{ZVICS}} \mbox{-} {\rm{IOCS}}}(87.24)$$

$${{\rm{Q}}}_{{\rm{sand}}}(0.0248\,{\rm{mg}}/{\rm{g}}) < {{\rm{Q}}}_{{\rm{IOCS}}}(0.083\,{\rm{mg}}/{\rm{g}}) < {{\rm{Q}}}_{{\rm{ZVICS}} \mbox{-} {\rm{IOCS}}}(0.131\,{\rm{mg}}/{\rm{g}})$$

**At pH 7**

$${{\rm{RF}}}_{{\rm{sand}}}(6.03) < {{\rm{RF}}}_{{\rm{IOCS}}}(13.78) < {{\rm{RF}}}_{{\rm{ZVICS}} \mbox{-} {\rm{IOCS}}}(67.33)$$

$${{\rm{Q}}}_{{\rm{sand}}}(0.013\,{\rm{mg}}/{\rm{g}}) < {{\rm{Q}}}_{{\rm{IOCS}}}(0.022\,{\rm{mg}}/{\rm{g}}) < {{\rm{Q}}}_{{\rm{ZVICS}} \mbox{-} {\rm{IOCS}}}(0.117{\rm{mg}}/{\rm{g}})$$

The pH_pzc_ values of ZVICS, IOCS and sand were 7.50, 7.00 and 5.00, respectively. A higher pHpzc resulted in a more positive charge on the sorption surface^49^, especially when the solution pH was acidic (pH 4). As a result, the sorption of As(V) onto mixed ZVICS and IOCS (ZVICS-IOCS) was better than that onto the IOCS and sand columns.

The sorption capacity of As(V) onto IOCS and ZVICS-IOCS in the current study were compared with the results of previous studies. To easily compare our results with other studies, we selected only iron-based sorbents, and the unit of sorption capacity is given as the mg/g of sorbent. Moreover, we also considered other studies with IOCS and iron-based sorbents (see Table 3).

**Table 3:** Comparison of various iron oxide and ZVI coated sands for As(V) removal (modified from Hsu *et al*.^23^ and Mohan and Pittman^58^).

Absorbent	pH	Experiment	Initial concentration of As(V), mg/L	Surface area (m^2^/g)	Flow (ml/min)	Adsorption isotherm	Sorption capacity (mg/g)	References
IOCS	7.6	Batch	0.325	5.1	—	Langmuir	0.018	Thirunavukkarasu *et al*.^16^
IOCS-2	7.6	Batch	0.10	—	—	Freundlich	0.008	Thirunavukkarasu *et al*.^17^
IOCS	7.6	Batch	0.10	10.6	—	Langmuir	0.043	Thirunavukkarasu *et al*.^18^
Iron hydroxide granulates (GIH)	7.0	Batch	5–100	—	—	Linear	5.2	Daus *et al*.^19^
Sulfate modified iron-oxide coated sand (SMIOCS)	4.0	Batch	0.5–3.5	3.74	—	Langmuir and Freudlich	0.128	Vashiya and Gupta^20^
							0.117	
							0.082	
	7.2							
	10.2							
Ferrihydrite	—	Batch	—	141	—	Freudlich	0.285	Thirunavukkarasu *et al*.^16^
Ferrihydrite coated sand	7.2	Batch	~75	—	—	—	0.202–0.483	Herbel and Fendorf^21^
Iron oxide coated cement (IOCC)	7.0	Batch	0.5–10	—	—	Langmuir	3.39–4.63	Kundu and Gupta^22^
IOCS	5	Batch	0.01–0.5	1.2	—	Langmuir	0.022	Hsu *et al*.^23^
IOCS	7	Batch	0.01–0.5	1.2	—	Langmuir	0.021	Hsu *et al*.^23^
IOCS	8.5	Batch	0.392	2.44	—	Langmuir	0.249	Garrido-Hoyos and Romero-Velazquez^24^
Iron hydroxide coated sand or iron hydroxide granules (GIH)	7.0	Column	0.5	—	18.6 ml/min.	—	2.3	Daus *et al*.^19^
Mixing of iron filling with sand	7.0 + 0.2	Column	0.44	—	16.67 ml/min	—	0.22–0.396	Leupin *et al*.^29^
Mixing of iron filling with sand	7.1–8.1	Column	0.5	—	16.67 ml/min	—	0.07–0.132	Mehta and Chaudhari^30^
IOCS	4 + 0.1	Column	10	0.78	17.2 ml/min	Freundlich	0.0825	This study
IOCS	7 + 0.2	Column	10	0.78	17.2 ml/min	Freundlich	0.022	This study
ZVICS-IOCS	4 + 0.1	Column	10	0.81	17.2 ml/min	Freundlich	0.131	This study
ZVICS-IOCS	7 + 0.2	Column	10	0.81	17.2 ml/min	Freundlich	0.117	This study

We compared our results with various IOCS and other oxide-coted sand materials from previous studies, as shown in Table 3. In general, the sorption capacity in the present study (column study) was 0.022–0.083 mg/g and 0.117–0.131 mg/g for IOCS and ZVICS-IOCS columns, respectively. Our results fell between 0.008 and 0.249 mg/g, which were obtained for IOCS in other studies^16–18,23,24^. However, those results were obtained from batch studies.

Based on the literature, it is difficult to directly compare other sorbents with coated sand media. However, we roughly compared our results with the results for other sorbents that were not IOCS and found that the sorption capacities of the sorbents varied from 0.082 to 5.2 mg/g (GIH) due to many different experimental conditions, such as pH, temperature, concentration range, sorbent dose, competitive ions, and source of treated water^16,19–22^.

When comparing only column experiments, granulated iron hydroxide (GIH) had a high sorption capacity (2.3 mg/g) because this sorbent is an iron hydroxide-coated sand consisting of an active-phase iron hydroxide content of 56–62% Fe_2_O_3_^19^, which is relatively high compared with those in IOCS (0.38% Fe_2_O_3_) and ZVICS (0.43% Fe_2_O_3_). As seen in Table 3, ZVICS-IOCS seems to be a good choice for As(V) removal from contaminated water. However, ZVICS has a lower surface area than the other sorbents; therefore, modification of the surface area of ZVICS can improve the efficiency of As removal.

### SEM analysis

**Figure 2:**
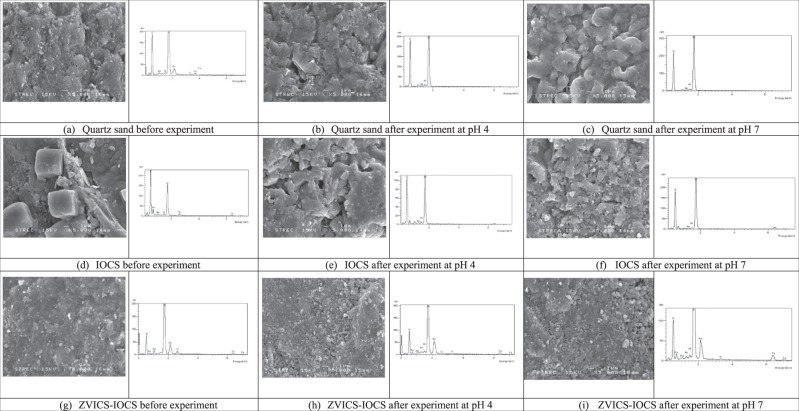
SEM images and the corresponding EDX spectra of the quartz sand before conducting column experiments (**a**) and after conducting column experiments at pH 4 (**b**) and pH 7 (**c**); of the IOCS before conducting column experiments (**d**) and after conducting column experiments at pH 4 (**e**) and pH 7(**f**); and of the ZVICS-IOCS before conducting column experiments (**g**) and after conducting column experiments at pH 4 (**h**) and pH 7 (**i**).

The SEM image was used to observe the surfaces of the reactive materials. The EDX/EDS analysis was used to explain the atomic distribution on the surfaces of the reactive materials. The SEM images and the corresponding EDX spectra of acid-washed natural sand, IOCS and ZVICS with IOCS before and after complete As(V) transport through columns under different conditions are shown in Fig. 2.

Fig. 2(a) shows the surface roughness of natural sand grains, and the sand surfaces after the completed transport tests at pH 4 and pH 7 are shown in Fig. 2(b,c), respectively. The results showed that the sand surfaces after the completed As(V) transport experiments at pH 4 and 7 had different surfaces than the natural sand before As(V) transport was conducted.

The image of the surface of IOCS shown in Fig. 2(d–f) illustrates that goethite is on the surfaces as concluded by XRD analysis, and in the form of cubic crystals (see Supplementary Information, Fig. SI.1). Lai *et al*.^50^ found that the pore size was increased by coating crystalline goethite on the sand’s surface. The larger specific surface area of IOCS (0.78 m^2^/g), which meant increased adsorption sites on the grain surface and enhanced chemical bonding, improved the As removal on IOCS. Therefore, sorption of As(V) onto IOCS was better than that of sand, which corresponded with the results of the retardation factor (*RF*) of IOCS. The sorption mechanism of As(V) on IOCS is that the Fe(III) oxide-coated sand sorbent is oxidized to FeOOH (hydrous ferric oxide), after which As(V) oxyanions are attracted to the IOCS and bound with active sites (–OH group).

Finally, As(V) oxyanions are bound to the IOCS surface and eliminate water molecules. The EDX spectra were collected at randomly selected points on the sorbent surface. The results from the corresponding EDX spectra show peaks of As(V) at both pH 4 and 7. This result confirms the sorption of As(V) onto the IOCS surface.

Figure 2(g–i) show SEM images of the combined ZVICS and IOCS column at pH 4 and 7, with some observable likely secondary corrosion products that have the characteristic of resembling boulder-like precipitates. The corrosion product was produced from Fe° corrosion, which caused the increase of As(V) removal by sorption and/or precipitation with the iron corrosion products^10,32,33^. The specific surface area of ZVICS (0.81 m^2^/g) was slightly higher than IOCS and quartz sand but the sorption capacity of ZVICS-IOCS was approximately 7 and 8.5 times greater than those of IOCS for pH 4 and pH 7, respectively (see Table 1). This may imply that the mixture of ZVICS and IOCS led to a higher reactive surface area for As(V) sorption and the production of more corrosion products, which induced an increase in the reactive surface of As(V). Moreover, the mixture of Fe° and IOCS showed a higher removal capacity for As(V) compared with IOCS alone^10^. The EDX analysis was used to evaluate the elemental content of the mixed ZVICS and IOCS column after uptake of As(V). The EDX spectra were collected from randomly selected points on adsorbent surfaces. The results indicated the presence of Fe at 3 peaks of energy of approximately 0.75 keV, 6.40 keV and 7.0 keV. In contrast, IOCS showed EDX spectra with only 2 peaks of Fe energy at approximately 0.75 keV and 6.40 keV. The studies of Prema *et al*.^49^ and Dada *et al*.^51^ showed that the EDX profile of nano zero-valent iron (nZVI) appeared to have Fe energy peaks at 0.8 keV, 6.4 keV and 7.1 keV. Therefore, the EDX spectra of ZVICS combined with IOCS give evidence that the random surface area consisted of iron oxide and zero-valent iron elements on the sand surface. According to the methodology, the coated sand samples were gold-coated prior to analysis, resulting in the appearance of a gold peak in some of the EDS spectra. Similarly, Sharma *et al*.^45^ found a gold (Au) peak in the spectrum due to gold coating of the coated sand prior to SEM analysis.

### Hydrus-1D model

**Figure 3:**
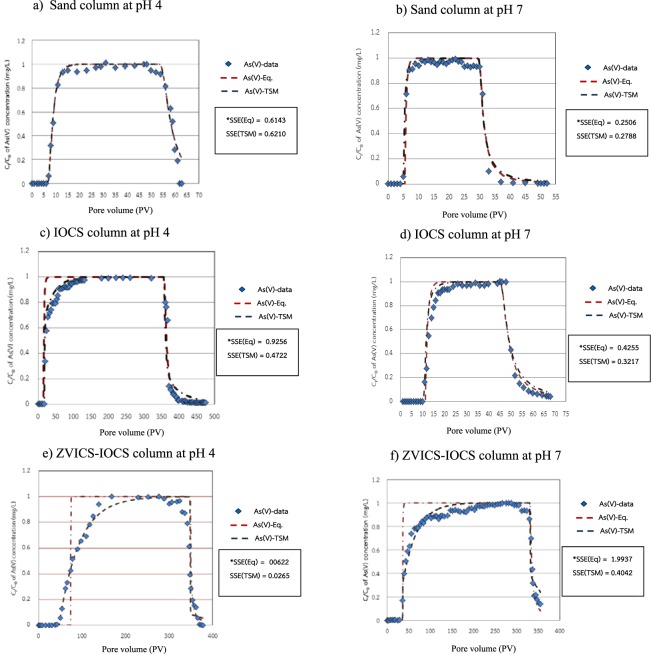
As(V) breakthrough curves for sand at pH 4 (**a**), sand at pH 7 (**b**), IOCS at pH 4 (**c**), IOCS at pH 7 (**d**), ZVICS-IOCS at pH 4 (**e**), and ZVICS-IOCS at pH 7 (**f**); Curve fitting of the column experiments and As(V) data with the equilibrium model (EQ) and nonequilibrium model (TSM) was produced by using the HYDRUS-1D model.

To understand the sorption mechanism during As(V) migration through different reactive materials under different pH conditions, the BTCs of As(V) were fitted by the uniform (equilibrium) model (EQ) and the two-site model (TSM), as the module in HYDRUS-1D, as shown in Fig. 3(a–f).

Figures 3(a,b) show that the fitted curves of the sand columns at pH 4 and 7 by the equilibrium transport model (EQ) were closer to the BTCs from the column experiments than the non-equilibrium model, with R^2^ values of 0.9763 and 0.9913, respectively, as shown in Table 4. Moreover, Table 4 shows the estimated transport parameters for As(V) breakthrough curves from the equilibrium convection-dispersion model (EQ) and two-site model (TSM) generated by the Hydrus-1D model. According to Table 4, The results of sum-of-square error (SSE) values (0.6143 and 0.2506, respectively) calculated from the equilibrium transport model in both the sand columns at pH 4 and 7 were less than those of the chemical non-equilibrium two-site model (0.6210 and 0.2788, respectively) (see Table 4), which indicates that most reactive sorption sites are mainly instantaneous sorption sites during As(V) transport through the sand column. Table 4 shows the calibrated parameters derived from HYDRUS-1D and reveals that the Freundlich constant (*Kf*) increased as sand column pH decreased. This agrees with the increased retardation factor (*RF*).

**Table 4:** Estimated transport parameters for As(V) breakthrough curves from the equilibrium model (EQ) and two-site model (TSM) generated by the Hydrus-1D model.

Column experiment (No.)	Reactive material	pH	Equilibrium model				Non-equilibrium model					
			*K*_*F*_	*1/n*	*SSE*	*R*^2^	*K*_*F*_	*1/n*	*f*	*α* (hr^−1^)	*SSE*	*R*^2^
1	Sand	4	9.81	0.58	0.6143	0.9763	9.50	0.59	0.99	0.0040	0.6210	0.9758
4	Sand	7	5.57	0.41	0.2506	0.9913	7.20	0.37	0.09	0.0038	0.2788	0.9894
2	IOCS	4	22.70	0.45	0.9256	0.9256	52.21	0.29	0.55	0.0006	0.4722	0.9759
5	IOCS	7	15.59	0.36	0.4255	0.9729	18.00	0.35	0.90	0.0050	0.3217	0.9805
3	ZVICS-IOCS	4	220.00	0.02	0.0622	0.8667	516.00	0.01	0.01	0.0003	0.0265	0.9182
6	ZVICS-IOCS	7	57.10	0.14	1.9937	0.9058	108.00	0.21	0.08	0.0006	0.4042	0.9717

*K_*F*_ and *1/n* are the Freundich constant (L^3^/M, -), ƒ the fraction of sorption sites, *α* is a first-order kinetic rate coefficient and SSE is sum of square error.

Figures 3(c–f) show fitted curves of IOCS at pH 4 and 7 and the ZVICS - IOCS combination at pH 4 and 7. The results of the fitted curves show that the two-site model (TSM) fitted curves were closer than the equilibrium model (EQ) with BTCs from the column experiments containing IOCS as reactive materials (column nos. 3–4); R^2^ values were 0.9759 and 0.9805 at pH 4 and 7, respectively. For ZVICS - IOCS as a reactive material, the two-site model (TSM) could explain the BTCs from the column experiments (column nos. 5–6) better than the equilibrium model, with R^2^ values of 0.9182 and 0.9717, respectively, as shown in Table 4. Moreover, the SSE values are shown in Table 4 and are summarized in terms of the comparison between TSM and EQ as follows:

**At pH 4**

$${{\rm{S}}{\rm{S}}{\rm{E}}}_{({\rm{T}}{\rm{S}}{\rm{M}})}\,{\rm{o}}{\rm{f}}\,{\rm{I}}{\rm{O}}{\rm{C}}{\rm{S}}\,{\rm{a}}{\rm{n}}{\rm{d}}\,\,({\rm{Z}}{\rm{V}}{\rm{I}}{\rm{C}}{\rm{S}}-{\rm{I}}{\rm{O}}{\rm{C}}{\rm{S}}) < {{\rm{S}}{\rm{S}}{\rm{E}}}_{({\rm{E}}{\rm{Q}})}\,{\rm{o}}{\rm{f}}\,{\rm{I}}{\rm{O}}{\rm{C}}{\rm{S}}\,{\rm{a}}{\rm{n}}{\rm{d}}\,({\rm{Z}}{\rm{V}}{\rm{I}}{\rm{C}}{\rm{S}}-{\rm{I}}{\rm{O}}{\rm{C}}{\rm{S}})$$

**At pH 7**

$${{\rm{S}}{\rm{S}}{\rm{E}}}_{({\rm{T}}{\rm{S}}{\rm{M}})}\,{\rm{o}}{\rm{f}}\,{\rm{I}}{\rm{O}}{\rm{C}}{\rm{S}}\,{\rm{a}}{\rm{n}}{\rm{d}}\,({\rm{Z}}{\rm{V}}{\rm{I}}{\rm{C}}{\rm{S}}-{\rm{I}}{\rm{O}}{\rm{C}}{\rm{S}}) < {{\rm{S}}{\rm{S}}{\rm{E}}}_{({\rm{E}}{\rm{Q}})}\,{\rm{o}}{\rm{f}}\,{\rm{I}}{\rm{O}}{\rm{C}}{\rm{S}}\,{\rm{a}}{\rm{n}}{\rm{d}}\,({\rm{Z}}{\rm{V}}{\rm{I}}{\rm{C}}{\rm{S}}-{\rm{I}}{\rm{O}}{\rm{C}}{\rm{S}})$$

The results indicate that the As(V) sorption onto IOCS and ZVICS - IOCS can be well described by TSM. Moreover, in each column, the fraction of instantaneous sorption sites (ƒ) was lower than 1 and the mass transfer value (*α*) had a smaller value, which led to less instantaneous sorption (or more kinetic sorption sites) and more pronounced tailing^41^. The fitted TSM breakthrough curves from the HYDRUS-1D model concerning the fraction of instantaneous sites (*f*) and the first kinetic sorption rate of As(V) onto IOCS at pH 4 and 7 (Fig. 3(c,d)) describe a better description of As(V) sorption onto IOCS, which indicates that As(V) could sorb onto both instantaneous sorption sites and kinetic sorption sites^24^. The Freundlich constant (*Kf*) decreased as pH increased from 4 to 7 for As(V) migration through the ZVICS-IOCS columns, which agrees with the decreased retardation factor (*RF*) from 37.18 to 13.78, respectively, (see Table 2) because at pH 4 the surface charge of IOCS became more positively charged. As(V) in oxyanionic forms, H_2_AsO_4_^−^ and H_2_AsO_4_^2−^ can sorb more strongly at pH 4 (lower than both PZCs of ~7.0–7.5) than at pH 7 in both IOCS and ZVICS-IOCS.

Moreover, the other Freundlich constant, *1/n*, appeared to decrease with decreasing pH, which implies the preferential sorption of As(V) at a lower pH (pH 4). The *1/n* values appeared to increase as the pH decreased, implying that under lower pH conditions, the sorption of As(V) seems to be more favorable^52^. This can be explained by the more heterogeneous surface of the sorbent at the lower pH.

The fraction of instantaneous sorption sites (*f*) is lower at lower pH, implying that the sorption mechanism of As(V) onto IOCS and ZVICS-IOCS has more nonequilibrium sorption sites (see Table 3). The instantaneous site fraction (*f*) was in the range of 55% to 90% for As(V) sorption onto IOCS at pH 4 and pH 7. The fraction of instantaneous sorption sites (*f*) of As(V) onto IOCS tended to increase by a factor of 2 as the pH increased from 4 to 7 because of the reduced negative charge on the IOCS surface (PZC_IOCS_ ~7.0). This is the reason why the mechanism of As(V) sorption/desorption onto IOCS at pH 7 is reversible or equilibrium sorption, as shown by the fact that the BTCs were more symmetrically shaped.

Similarly, the results of the fitted TSM curves from the HYDRUS-1D model onto the mixture of ZVICS and IOCS columns under different pH conditions well depicted a calculated BTC fitted to the experimental data (Fig. 3(e,f)). The instantaneous site (*f*) was in a range between 1% to 8% for As(V) onto IOCS at pH 4 and pH 7. Compared with those of IOCS, the fraction of instantaneous sorption sites (*f*) of As(V) onto ZVICS dramatically decreased because secondary corrosion products may have been generated. Mak *et al*.^53^ used a mixture of Fe° and IOCS in column experiments, and the results confirmed that the deposited iron corrosion products had an inner-sphere morphology and were deposited onto the IOCS surfaces, causing an increase in retention time and higher removal capacity of As(V) compared to the results for IOCS column only^54^. More iron corrosion products appeared to be deposited on the IOCS than on the original IOCS, implying that the kinetic sorption sites were increased and the retention times of As(III) and Cr(VI) in the mixed column were increased. As mentioned above, the results were in agreement with the fitted parameter, the fraction (*f*) of equilibrium sites, which appeared to be lower for the ZVICS - IOCS system than for the IOCS system at the same pH (see Table 3). The high proportion of kinetic sorption sites (1-*f*) implies that As(V) sorption onto ZVICS + IOCS seems to follow a more nonequilibrium sorption mechanism, which is why the BTCs of these columns clearly show a tailing phenomenon^39^. Moreover, the removal efficiency of As(V) in the ZVICS + IOCS columns was approximately 60 and 83 percent at pH 4 and 7, respectively, which confirms that the sorption/desorption behavior of As(V) onto the ZVICS-IOCS columns tends to be more nonequilibrium sorption as pH decreases and implies that the sorption of As(V) onto the column at pH 4 was stronger than that at pH 7.

The kinetic rate (*α*) was influenced by the pH and appeared to increase with increasing pH for IOCS and ZVICS. The slower sorption rate might be caused by an increase in the deposited iron corrosion generated in the IOCS and ZVICS-IOCS systems at the lower pH, implying a slow diffusion process through the deposited layers. Moreover, at the same pH, the rate coefficient of the ZVICS-IOCS system seemed to have lower values because the thickness of the deposited iron corrosion products was thicker than that of the IOCS (see Table 4). This agreed with the study of Mak *et al*.^10^ who found that the sorption of As(V) onto IOCS and a combination of IOCS and ZVI in batch experiments at an initial pH of 7 was characterized by kinetic behavior in which the amount of As(V) sorbed onto the sorbents increased with time. They measured the amount of As(V) on the IOCS and found that As(V) sorption increased from 130.2 to 175.7 µg/sample from 10 min to 60 min, indicating that As(V) sorption on IOCS occurred kinetically, and that the sorption of As(V) onto the iron corrosion products was 2.5 times higher at 60 min than that at 10 min due to the continuous increase in iron corrosion products.

Furthermore, Mak *et al*.^53^ found that ZVI contributed a large amount towards As(V) removal via sorption/coprecipitation with iron corrosion products^55^, which is a time-dependent sorption mechanism. Moreover, the deposited iron corrosion generated in IOCS can remove As(V) from solution, and Mak *et al*.^53^ found that the deposited layers on IOCS in columns with a combination of Fe° and IOCS without humic acid were 3 times thicker than the iron oxide layer of IOCS.

Similarly, Lai *et al*.^50^ studied the sorption characteristics in a system consisting of iron-coated sands and As solution at a pH of 2.5 in batch tests. The kinetic results showed that the sorption rate of As(V) on IOCS was initially fast and then gradually slowed and approached equilibrium. Moreover, the pseudo-second order kinetic adsorption model fitted well the kinetic sorption reactions of As(V) onto IOCS, and the equilibrium sorption capacity (q_e_) ranged from 0.94 to 3.57 mg/g. The kinetic results of As(V) sorption onto IOCS at pH 7 revealed that the initial removal efficiency of As(V) decreased as the pH increased from pH 5 to pH 8 and the equilibrium time was approximately 8 h^23^. In addition, the study of Oblonsky *et al*.^56^ revealed that the sorption of As(V) onto iron oxides is dominated by surface complexation mechanisms, which involve passivation as a layer onto Fe° and the study of Su and Puls^55^ found that As(V) sorption onto ZVI at pH 7–9 proceeded via kinetic processes involving surface complexation mechanisms. Similarly, the results of TSM-fitted curves from the HYDRUS-1D model and the kinetic sorption isotherm could imply that in the mechanism of As(V) sorption onto IOCS, the sorption sites were divided into instantaneous and kinetic sorption sites. Because of the heterogeneous sorbent surface, sorption to some surfaces would be non-equilibrium sorption sites^43^. Sun *et al*.^57^ studied the removal of As(V) and As(III) by using ZVI powder with ZVI concentration of 2.5 g/L under anaerobic and aerobic conditions. For the two As concentrations of this experiment, the removal processes of both As(V) and As(III) conformed to first-order kinetics. On the other hand, their experiment found that As in both As(V) and As(III) forms could be removed more efficiently under aerobic conditions, especially As(V), which could be attributed to the sorption of As onto iron and its corrosion products because of the interaction between As compounds and iron oxyhydroxides (FeOOH). Therefore, the results of these studies may confirm the result of As(V) sorption onto the ZVICS-IOCS combination TSM-fitted curves using the HYDRUS-1D model. The results of kinetic sorption could confirm that the sorption sites could be divided into two types of sorption.

## Conclusions

In conclusion, As(V) sorption onto different reactive materials under acidic and neutral conditions was investigated in column experiments. As(V) removal increased with decreasing pH. It is suggested that the removal of As(V) increased with decreasing pH because the pHpzc values of sand, IOCS and ZVICS- IOCS were higher than the acidic pH of pH 4, which led to a positive charge on the surfaces and then to the higher removal capacity of As(V) on these reactive materials, mainly through electrostatic sorption. The pore size and specific surface area were increased by coating the sand surface with crystalline goethite, which led to higher sorption capacity of As, whereas the ZVICS- IOCS combination may have shown some observable secondary corrosion products, which caused an increase in the reactive surface for As(V); this is why the mixture of ZVICS and IOCS showed the highest removal capacities of As(V) compared with IOCS alone. The mechanism of As(V) sorption onto sand at pH 4 and pH 7 corresponded to the uniform (equilibrium) solute transport model (EQ), whereas those of IOCS and ZVICS-IOCS sorption corresponded to the chemical nonequilibrium two-site model (TSM). In addition, for IOCS and ZVICS, the kinetic rate (*α*) was increased as increasing the pH increased, and the fraction of instantaneous sorption sites (*f*) sharply decreased compared with the original sand media, particularly for ZVICS-IOCS. This might be due to the generation of secondary corrosion products in the IOCS. The findings from this study support the use of efficient reactive materials to capture As in contaminated groundwater, specifically, the combination of ZVICS with IOCS, which had the highest sorption capacity and retardation factor (*RF*). Moreover, the results from the mathematical model could be further applied for remediation design at the pilot scale at contaminated sites.

## Supplementary information

Supplementary information.

## References

[1] Nova scotia environment, The drop on water Arsenic, http://www.evowater.ca/uploads/arsenic_nova_scotia_well_water.pdf, (accessed 20/August/2017).

[2] WHO. Guidelines for drinking water quality, Vol 1: Recommendations. 2nd ed. World Health Organization: Geneva (1993).

[3] Choprapawon, C. & Ajjimangkul, S. Major interventions on chronic arsenic poisoning in Ronpibool District, Thailand—Review and Long-Term Follow Up, in W.R. Chappel, C.O. Abernathy, R.L. Calderon (eds.), *Arsenic Exposure and Health Effects III*, San Diego, California. 355–362 (1999).

[4] Weerasiri T, Wirojanagud W, Srisatit T (2013). Arsenic contaminantion in soils, water and plants surrounding gold mind at Wangsaphung, Loei province, Thailand. J. Environ. Res. Develop..

[5] Bashkin VN, Wongyai K (2002). Environmental fluxes of arsenic from lignite mining and power generation in northern Thailand. J. Environ. Geol..

[6] Kim KW, Chanpiwat P, Hanh HT, Phan K, Sthiannopkao S (2011). Arsenic geochemistry of groundwater in Southeast Asia. Front. Med..

[7] Pansamut S, Wattayakorn G (2010). Arsenic contamination in water from Suphan buri province, Thailand. J. Environ. Res. Develop..

[8] Williams M (2001). Arsenic in mine waters: an international study. J. Environ. Geol..

[9] Obiri-Nyarko F, Grajales-Mesa SJ, Malina G (2014). An overview of permeable reactive barriers for *in situ* sustainable groundwater remediation. J. Chemosphere..

[10] Mak, M. S., Lo, I. M. & Liu, T. Synergistic effect of coupling zero-valent iron with iron oxide-coated sand in columns for chromate and arsenate removal from groundwater: Influences of humic acid and the reactive media configuration. *J. Water Res*. **45**, 6575–6584 (2011a). 10.1016/j.watres.2011.10.002 22018698

[11] Henderson AD, Demond AH (2007). Long-term performance of zero-valent iron permeable reactive barrier: a critical review. J. Environ. Eng. Sci..

[12] Kamolpornwijit W, Liang L, West OR, Moline GR, Sullivan AB (2003). Preferential flow path development and its influence on long-term PRB performance: column study. J. Contam. Hydrol..

[13] Zhang Y, Gillham RW (2005). Effects of gas generation and precipitates on performance of Fe° PRBs. J. Ground Water..

[14] Guan X (2015). The limitations of applying zero-valent iron technology in contaminants sequestration and the corresponding countermeasures: The development in zero-valent iron technology in the last two decades (1994–2014). J. Water Res..

[15] Dhagat, A., Goyal, B. & Sailo, L. Effect of Size of Iron Oxide Coated Sand (IOCS) on Removal of Cr (VI) from Water. In the Proceedings on Ecological, Environmental and Biological Sciences (ICEEBS’2013), Singapore. 316–320 (2013).

[16] Thirunavukkarasu OS, Viraraghavan T, Subramanian KS (2001). Removal of arsenic in drinking water by iron oxide-coated sand and ferrihydrite-batch studies. Water Qual. Res. J. Can..

[17] Thirunavukkarasu OS, Viraraghavan T, Subramanian KS, Tanjore S (2002). Organic arsenic removal from drinking water. Urban. Water.

[18] Thirunavukkarasu, O. S. Viraraghavan, T. & Subramanian, K. S. Arsenic removal from drinking water using iron-oxide coated sand. *J. Water, Air, Soil Pollut*. **142**, 95–111 (2003).

[19] Daus B, Wennrich R, Weiss H (2004). Sorption materials for arsenic removal from water: a comparative study. J. Water Res..

[20] Vaishya RC, Gupta SK (2004). Modeling Arsenic(V) Removal from Water by Sulfate Modified Iron‐Oxide Coated Sand (SMIOCS). J. Sep. Sci. Technol..

[21] Herbel M, Fendorf S (2006). Biogeochemical processes controlling the speciation and transport of arsenic within iron coated sands. J. Chem. Geol..

[22] Kundu S, Gupta AK (2006). Investigation on the adsorption efficiency of iron oxide coated cement (IOCC) towards As(V) – kinetics, equilibrium and thermodynamic studies. Colloids Surf. A: Physicochem. Eng. Asp..

[23] Hsu JC, Lin CJ, Liao CH, Chen ST (2008). Removal of As(V) and As(III) by reclaimed iron-oxide coated sands. J. Hazard. Mater..

[24] Garrido-Hoyos S, Romero-Velazquez L (2015). Synthesis of minerals with iron oxide and hydroxide contents as sorption medium to remove arsenic from water for human consumption. Int. J. Environ. Res. Public. Health.

[25] Trois C, Cibati A (2015). South African sands as an alternative to zero valent iron for arsenic removal from an industrial effluent: Batch experiment. J. Environ. Chem. Eng..

[26] Das S, Lindsay MB, Essilfie-Dughan J, Hendry M (2017). J. Dissolved selenium(VI) removal by zero-valent iron under oxic conditions: influence of sulfate and nitrate. ACS Omega..

[27] Ruhl AS, Jekel M (2014). Degassing, gas retention and release in Fe(0) permeable reactive barriers. J. Contam. Hydrol..

[28] Moraci N, Ielo D, Bilardi S, Calabro PS (2015). Modelling long term hydraulic conductivity behavior of zero valent iron column tests for PRB design. Can. Geotech. Journal..

[29] Leupin OX, Hug SJ, Badruzzaman ABN (2005). Arsenic removal from simulated groundwater using household filter columns containing iron filings and sand. J. Environ. Sci. Technol..

[30] Mehta VS, Chaudhari SK (2015). Arsenic removal from simulated groundwater using household filter columns containing iron filings and sand. J. Water Process. Eng..

[31] Moraci N, Ielo D, Bilardi S, Calabro PS (2017). Modelling long term hydraulic conductivity behavior of zero valent iron column tests for PRB design. Can. Geotech..

[32] Li S (2019). High dispersions of nano zero valent iron supported on biochar by one-step carbothermal synthesis and its application in chromate removal. RSC Adv..

[33] Van Raij B, Peech M (1972). Electrochemical properties of some oxisols and alfisols of the tropics. Soil. Sci. Soc. Am. Pro..

[34] Brunauer S, Emmett PH, Teller E (1938). Adsorption of gases in multi molecular layers. J. Am. Chem. Soc..

[35] Wikiniyadhanee R, Chotpantarat S, Ong SK (2015). Effects of kaolinite colloids on Cd2+ transport through saturated sand under varying ionic strength conditions: Column experiments and modeling approaches. J. Contam. Hydrol..

[36] Masipan T, S. Chotpantarat S, Boonkaewwan S (2016). Experimental and modelling investigations of tracer transport in variably saturated agricultural soil of Thailand: Column study. Sustain. Environ. Res..

[37] Chotpantarat S, Kiatvarangkul N (2018). Facilitated transport of cadmium with montmorillonite KSF colloids under different pH conditions in water-saturated sand columns: Experiment and transport modeling. J. Water Res..

[38] Waleeittikul A, Chotpantarat S, Ong SK (2019). Impacts of salinity level and flood irrigation on Cd mobility through a Cd-contaminated soil, Thailand: experimental and modeling techniques. J. Soil. Sediment..

[39] Chotpantarat S, Sutthirat C, Ong SK, Osathaphan K (2011). Effect of pH on transport of Pb2+, Mn2+, Zn2+ and Ni^2+^ through lateritic soil: Column experiments and transport modeling. J. Environ. Sci..

[40] Maraqa MA (2001). Effects of fundamental differences between batch and miscible displacement techniques on sorption distribution coefficient. J. Environ. Geol..

[41] Šimůnek J, van Genuchten MT (2008). Modeling Nonequilibrium Flow and Transport Processes Using HYDRUS. Vadose Zone J..

[42] Selim, H. M., Davidson, J. M., & Mansell, R. S. Evaluation of a two-site adsorption–desorption model for describing solute transport in soil. Proc. Summer Computer Simulation Conf., Washington, DC. 12–14 July, p. 444–448 (1976).

[43] Van Genuchten MT, Wagenet RJ (1989). Two-site/two-region models for pesticide transport and degradation: Theoretical development and analytical solutions. Soil. Sci. Soc. Am. J..

[44] Bakather, O. Y., Kayvani Fard, A., Khraisheh, M., Nasser, M. S., & Atieh, M. A. Enhanced adsorption of selenium ions from aqueous solution using iron oxide impregnated carbon nanotubes. *J. Bioinorg. Chem. Appl*. 4323619 (2017). 10.1155/2017/4323619 PMC5438866 28555093

[45] Sharma SK, Petrusevski B, Schippers JC (2002). Characterisation of coated sand from iron removal plant. Water Sci. technology: water supply..

[46] Genç-Fuhrman H, Bregnhøj H, McConchie D (2005). Arsenate removal from water using sand–red mud columns. J. Water Res..

[47] Gupta VK, Saini VK, Jain N (2005). Adsorption of As(III) from aqueous solutions by iron oxide-coated sand. J. Colloid interface Sci..

[48] Ko I, Davis AP, Kim JY, Kim KW (2007). Arsenic removal by a colloidal iron oxide coated sand. J. Environ. Eng..

[49] Prema P, Thangapandian S, Selvarani M, Subharanjani S, Amutha C (2011). Color removal efficiency of dyes using nanozerovalent iron treatment. J. Toxicol. Environ. Chem..

[50] Lai CH, Chen CY, Wei. BL, Yeh SH (2002). Cadmium adsorption on goethite coated sand in the presence of humic acid. J. Water Res..

[51] Dada, A. O., Adekola, F. A. & Odebunmi, E. O. Synthesis and characterization of iron nanoparticles and its ash rice husk supported nanocomposite. In Proceedings of the 1st African international conference/workshop on application of nanotechnology to energy, health and environment, UNN, March 23–29 (2014).

[52] Silva MMF (2012). Adsorption of an industrial anonic dye by modified-KSFmontmorillonite: evaluation of the kinetic, thermodynamic and equilibrium data. Chem. Eng. J..

[53] Mak MS, Rao P, Lo IM (2011). Zero-valent iron and iron oxide-coated sand as a combination for removal of co-present chromate and arsenate from groundwater with humic acid. J. Environ. Pollut..

[54] Noubactep C (2008). A critical review on the process of contaminant removal e0-H_2_O systems. Environ. Technol..

[55] Su C, Puls R (2001). Arsenate and arsenite removal by zerovalent iron: effects of phosphate, silicate, carbonate, borate, sulfate, chromate, molybdate, and nitrate, relative to chloride. J. Environ. Sci. Technol..

[56] Oblonsky LJ, Ryan MP, Isaacs HS (2000). *In situ* XANES study of the formation and reduction of the passive film formed on Fe in acetate solution. J. Corros. Sci..

[57] Sun H, Wang L, Zhang R, Sui J, Xu G (2006). Treatment of groundwater polluted by arsenic compounds by zero valent iron. J. Hazard. Mater..

[58] Mohan, D. & Pittman, C. U. Arsenic removal from water/wastewater using adsorbent – a critical review. *J. Hazard. Mater.* **142**, 1–53 (2007). 10.1016/j.jhazmat.2007.01.006 17324507

