# Supplementary material for: Influences of pH on transport of arsenate (As5+) through different reactive media using column experiments and transport modeling
Source: Sci Rep. 2020 Feb 26;10:3512. doi: 10.1038/s41598-020-59770-1 (PMC7044194; doi:10.1038/s41598-020-59770-1)
Supplement: Supplementary file 1 — Supplementary information. [file 41598_2020_59770_MOESM1_ESM.docx]

**Supplementary Information**

**Influences of pH on transport of arsenate (As5+) through different reactive media using Column experiments and transport modeling**

Srilert Chotpantarat ^1,3,4^* and Chonnikarn Amasvata^2^

^1^Department of Geology, Faculty of Science, Chulalongkorn University, Bangkok 10330, Thailand.

^2^International Postgraduate Programs in Environmental Management, Graduate School,

Chulalongkorn University, Bangkok 10330, Thailand.

^3^Research Program of Toxic Substance Management in the Mining Industry, Center of Excellence on Hazardous Substance Management (HSM), Chulalongkorn University, Bangkok, Thailand

**^4^** Research Unit of Green Mining (GMM), Chulalongkorn University, Bangkok, Thailand

***Corresponding author**. E-mail address: [csrilert@gmail.com](mailto:csrilert@gmail.com); Tel.: +66-2218-5455;

fax: +66-2218-5464.

**Goethite (α-FeO(OH))**

**Quartz alpha(SiO_2_)**

**Figure SI.1** X-ray Diffraction pattern of synthesis IOCS

**Iron synthesis**

**(Fe)**

**Iron Silicide (Fe_2_Si)**

**Quartz alpha(SiO_2_)**

**Figure SI.2** X-ray Diffraction pattern of synthesis ZVICS
